# Innovation through Tradition: The Current Challenges in Cancer Treatment

**DOI:** 10.3390/ijms23105296

**Published:** 2022-05-10

**Authors:** Luigi Sapio, Silvio Naviglio

**Affiliations:** Department of Precision Medicine, University of Campania “Luigi Vanvitelli”, L. De Crecchio 7, 80138 Naples, Italy

Despite the huge efforts in identifying novel risk factors, earlier diagnostic markers and alternative therapeutic approaches, malignant disorders continue to pose the second leading cause of death worldwide [1]. Regrettably, this tendency does not appear to reverse its course in the near future, leaving an open matter to the cancer-related implications. Two main troubles urge the scientific community towards the research of additional strategies to effectively improve cancer management, namely an ethical and moral imperative driven by the Hippocratic oath and an economical one, due to their increasing financial impact on national healthcare systems [2].

Whilst the achieved therapeutic advances have raised both progression-free and overall survival, there is still a great deal to be done in making cancer progressively curable. Contrary to the previous expectation, indeed, malignancies are not fully turning from life-threatening to chronic diseases, even though it may be appropriate to refer to a specific cancer phase with long-lasting features [3]. Except for a subset of tumor patients who successfully responds to standard treatments, a repetitive cycle of remission and recurrence actually occurs in most circumstances, assuming distinctive chronic peculiarities in this timeframe. A completely different matter applies for advanced and metastatic tumors at onset, in which palliative care is typically administrated with the purpose of alleviating symptom burden and spiritual and psychological distress rather than treating cancer [4].

In view of the abovementioned scenario, this Special Issue (SI) has been conceived to facilitate the dissemination of the latest research and up-to-date review articles on interesting findings in cancer treatment and expectations. According to the aim and scope of the *IJMS* journal, the emphasis has mainly been placed on molecular studies in biology, chemistry and medicine field.

Because a considerable number of anticancer drugs are naturally occurring molecules or designed following the natural ones, special attention has been paid to this research area [5]. Not surprisingly, three out of seven published studies are based on extracted or synthetically derived natural compounds.

Latypova and colleagues designed and developed a series of heterocyclic compounds containing a spiro-fused pyrrolo [3,4-a]pyrrolizine and tryptanthrin framework [6]. They observed that cycloadducts impaired the growth of both human erythroleukemia (K562) and human cervical carcinoma (HeLa) cells, arresting in the G2/M phase and inducing apoptosis. Moreover, they also provided convincing evidence of these compounds’ ability to inhibit cell motility and change the actin cytoskeleton structure of tumor cells. Even though additional studies are absolutely needed to define the exact cellular ways, these results assume that a spiro-fused pyrrolo [3,4-a]pyrrolizine and tryptanthrin structure may be considered a new promising pharmacophore unit for the development of additional cancer-related drugs.

Apart from corroborating the antineoplastic feature mediated by Chlorogenic Acid (CGA) [7,8], Salzillo and coworkers recognized this compound as a potential partner in Doxorubicin-based therapy, expressly in human osteosarcoma cells (OS) [9]. Compared to anthracycline alone, they displayed a further decline in cell viability and growth, as well as in clonogenic potential, when CGA was administered in combination with Doxorubicin in U2OS and MG-63 OS cells. CGA’s ability to enhance Doxorubicin-mediated cytotoxic effects was also accompanied by a significant rise in apoptosis occurrence. However, even more interestingly, analogous treatments carried out in H9c2 rat cardiomyocyte cells ameliorated Doxorubicin-induced toxicity, thus suggesting concomitant cardioprotective outcomes exerted by this natural molecule. Mechanistically, the inactivation of p44/42 MAPK was detected in response to combination treatments, while the PD98059-mediated p44/42 MAPK impairment further deteriorated OS cell growth.

As a relevant target in anticancer therapy, human topoisomerase IB (hTopIB) is involved in several cellular processes affecting DNA homeostasis, such as cleaving and binding when supercoiling occurs [10]. In their review, Ottaviani et al. collected the existing cancer evidence concerning the natural product’s ability to target and inactivate hTopIB [11]. Besides analyzing the state-of-the-art camptothecin (CPT) derivates, as well as those arising from indenoisoquinoline compounds, the authors drew attention to the marine and Antarctic worlds as potential sources of additional natural hTopIB inhibitors. Thanks to their unique chemical structure, aquatic-derived hTopIB inhibitors may overcome drug resistance induced by intense and prolonged treatments of CPT derivatives, representing an alternative choice in cancer management. However, this latter assumption is intended as a long-term objective by authors, given that the development of selective and effective anticancer agents demands supplementary studies.

Three additional works published within the SI belong to review articles, and although they debate well-known perspectives of cancer disease, a critical and constructive analysis of specific topics and matters is being provided.

Sun and colleagues, for instance, examined the mechanisms of interaction between chemotherapeutic agents and cell cycle regulators in cancer treatment [12]. Outlining the potential challenges and perspectives in combining together these two drug classes, the authors emphasized how an artificial regulation of the cell cycle could synergically affect the chemotherapy outcome. However, they did not rule out the therapeutic benefit of adjuvating surgery or radiotherapy with cell-cycle-specific drugs. Debating the potential clinical carriers, the authors lastly proposed nano-drug delivery system (NDDS) as a useful tool to improve the effectiveness of cell cycle regulators, expressly in combination with chemotherapeutic agents.

The high versatility of NDDS could also enable the application of future challenges in cancer therapy, such as improving the cascade technology treatments [13]. Despite both pharmacological approaches achieving satisfactory outcomes in preclinical models, human trials reported conflicting results due to either tumor or microenvironment heterogeneity. Therefore, combining nanocarriers with cascade-targeting ability can damage tumor cells or specific organelles more precisely, improving killing skills and reducing adverse reactions.

The crucial role of the microenvironment in sustaining tumor growth and progression is also remarked by Munir and coworkers, who extensively addressed tumor-associated macrophages (TAMs) from a therapeutic point of view in breast cancer [14]. As a major cellular component in the tumor microenvironment, the authors summarized TAMs origin, mechanisms of recruitment and contributions to tumor progression. Finally, they also explored the possibility of using miRNAs and exosomes to retrain TAMs in breast cancer therapeutically.

As claimed in the launching campaign, innovative technological and methodological tools aimed at providing insight into cancer comprehension and treatment have been considered of interest for our SI. Proof of this assumption is the original article published by Schmitz and co-workers, which recognized the murine cell line L929 as an in vitro system for the rapid assessment of Methionine restriction (MetR). Using liquid chromatography and mass spectrometry analysis, they defined a metabolic fingerprint and identified metabolites representing normal or MetR conditions, thus proposing the L929 cell model as a rapid and efficient in vitro assay for testing potential cancer metabolic targets.

As guest academic editors of this SI, we are quite enthused by the reception obtained in terms of publication and dissemination. Along with conceptual and technical innovative approaches, interesting insight into our current knowledge and understanding of cancer is provided within the printed articles.

Treating malignancies have always been accounted as one of the most arduous challenges for the scientific community. Nevertheless, it is undeniable that, albeit in small steps, considerable progress has been made in cancer therapy over the years. Although the vast majority of the topics covered in this SI are far away from clinical practice, we earnestly hope that these findings could spark new ideas in making more treatable cancer diseases in future.
